# RFID-Based Real-Time Salt Concentration Monitoring with Adaptive EKF

**DOI:** 10.3390/s25123826

**Published:** 2025-06-19

**Authors:** Renhai Feng, Xinyi Lin

**Affiliations:** 1 School of Electrical and Information Engineering, Tianjin University, Tianjin 300072, China; 2 School of Physics and Electronic Engineering, Xinjiang Normal University, Urumqi 830054, China; 3 Xinjiang Key Laboratory of Luminescence Minerals and Optical Functional Materials, Urumqi 830054, China

**Keywords:** radio frequency identification (RFID), Cole–Cole model, adaptive extended Kalman filter (AEKF), concentration monitoring

## Abstract

Salt concentration monitoring is crucial for industrial process control and wastewater management, yet existing methods often lack real-time capability or require invasive sampling. This paper presents a novel RFID wireless sensing system for noninvasive solution concentration monitoring, combining physical modeling with advanced estimation algorithms. By combining the Cole–Cole model and the slit cylindrical capacitor (SCC) model, the system establishes physics-based state-space models to characterize concentration-dependent RFID signal variations. The concentration dynamics are modeled as a hidden Markov process and tracked using an adaptive extended Kalman filter (AEKF). The AEKF algorithm avoids computationally expensive inversion of complex observation equations while automatically adjusting noise covariance matrices via innovation sequence. Experimental results demonstrate a mean relative error (MRE) of 2.8% for CaCl_2_ solution across 2–10 g/L concentrations. Within the experimentally validated optimal range (2–8 g/L CaCl_2_), the system maintains MRE below 3% under artificially introduced measurement noise, confirming its strong robustness. Compared with baseline approaches, the proposed AEKF algorithm shows improved performance in both accuracy and computational efficiency.

## 1. Introduction

Rapid industrialization has led to a significant increase in industrial wastewater discharge, particularly high-salinity wastewater containing total dissolved inorganic salts exceeding 3.5% [1]. Saline wastewater dischargement is not only against environmental protection but also a waste of potential salt resources. Since optimal treatment efficiency requires customized approaches for different salinity levels [2], accurate concentration monitoring prior to treatment becomes critically important for both environmental protection and resource recovery.

At present, a variety of high-precision analytical methods have been developed in the field of solution concentration detection. Wen et al. established and validated an ion chromatography method for determining sodium, potassium, magnesium, calcium, and chloride concentrations in parenteral nutrition solutions [3], yet this approach faces challenges of high instrument costs and complex sample pretreatment requirements. Grabarczyk et al. reviewed the application of electrochemical methods for tin detection in environmental samples [4], but their industrial implementation is hindered by electrode susceptibility to wastewater corrosion and frequent calibration. Ma et al. employed near-infrared (NIR) spectroscopy to measure soil salinity concentrations [5]. Despite their capability for accurate quantification at trace concentration levels, the latency and economic constraints of these methods limit their industrial applications. Consequently, there is an urgent need for a nondestructive detection technology that offers reasonable costs, real-time processing capabilities, and compatibility with industrial environments.

In recent years, the development of radio frequency identification (RFID) has created a new approach to sensor development [6,7]. Zhou et al. presented a method of measuring chloride ion concentration in concrete based on RFID tags [8]. Makarovaite et al. used ultra-high-frequency RFID tags to monitor aqueous and organic liquids [9]. Qian et al. used RFID to monitor minerals in water, reflecting ion concentrations through changes in tag capacitance [10]. Compared with other noninvasive methods, RFID sensing systems are cost-effective and easy to deploy, making them a preferred noninvasive option. RFID sensor tags are thin and flexible, easy to install, and do not come into contact with the sample under test. However, current research exhibits a notable gap in addressing saline wastewater monitoring, particularly concerning inorganic salt concentration monitoring. Therefore, this paper proposes a method for salt concentration monitoring using RFID and establishes a physical model between RFID signals and concentrations.

Although RFID sensing systems offer significant advantages, they also face some limitations. The response of the RFID tag to the salt concentration is nonlinear, and the target concentration cannot be directly derived from raw measurements. Thus, a robust method is required for real-time state estimation. Dynamic Bayesian Network (DBN) [11] and support vector machine (SVM) [12] methods involve extensive computation and experimentation to identify optimal parameters. Improper parameters or structures can result in degraded or unstable network performance. Particle filters (PF) [13] have high computational complexity, especially in the case of high-dimensional space or a large number of particles. The Hidden Markov Model (HMM) [14] is a probabilistic timing model based on the Markov hypothesis, which states that the present state is solely determined by the preceding instant. The Kalman filter (KF) [15] is an optimal recursive algorithm that estimates system states through noisy observations, but it remains constrained to linear systems. On this basis, the temporal evolution of concentration is conducted as a continuous hidden Markov process, where the concentration dynamics are governed by stochastic transitions. Since the observation model is nonlinear and with noise, we design an improved extended Kalman Filter (EKF) for efficient state estimation. The EKF locally linearizes the observations and recursively updates the concentration estimates, enabling accurate tracking of continuous concentration changes from noisy measurements.

This paper establishes an RFID sensing system for concentration estimation and proposes an adaptive EKF (AEKF) for tracking solute concentration. The main contributions are as follows.

- Noninvasive Dual-tag RFID Sensing System with Physics-based Modeling: We propose a novel dual-tag RFID wireless sensing system that enables noninvasive concentration detection. Based on the Cole–Cole model, we establish accurate state and observation models that fundamentally characterize the system dynamics, overcoming the limitations of conventional empirical approaches. This physics-based modeling provides a solid foundation for the subsequent algorithmic estimation.
- Forward Estimation Framework based on KF: Conventional concentration estimation methods require computationally intensive inversion of the complex observation model z=h(x) to obtain the state *x* from observation *z*, making them impractical for real-time monitoring applications. The proposed KF-based approach fundamentally transforms this inverse problem by utilizing only forward calculations of z=h(x) within its recursive prediction and update mechanism. This avoids the need for iterative equation solving while maintaining estimation accuracy through analytical state updates via the Kalman gain matrix, enabling real-time operation without compromising precision.
- Dynamic Noise Adaptation Using Innovation Sequence: Existing EKF-based approaches often assume fixed noise covariances (Q and R), leading to degraded performance in real-world environments. The proposed AEKF iteratively updates Q and R matrices using innovation sequence, enabling automatic adaptation to time-varying noise conditions. This adaptation mechanism significantly improves estimation stability in noisy RFID sensing scenarios without requiring manual parameter tuning.

The rest of this paper is structured as follows. Section 2 details the operating principles of the RFID sensing system and presents the system modeling framework, including the proposed concentration tracking algorithm. Section 3 describes the experimental process and results, and provides performance analysis. Section 4 analyzes the experimental results, discusses the limitations of the research, and explores potential future research directions. Finally, Section 5 summarizes the content of the full text.

## 2. Materials and Methods

### 2.1. RFID Sensing System

Direct measurement of relative permittivity in liquid using a professional network analyzer is invasive. Due to the corrosive nature of saline wastewater, it is not feasible to directly measure the dielectric properties of such liquids. To solve the problems mentioned above, a noninvasive system based on dual RFID is proposed.

**Figure 1:**
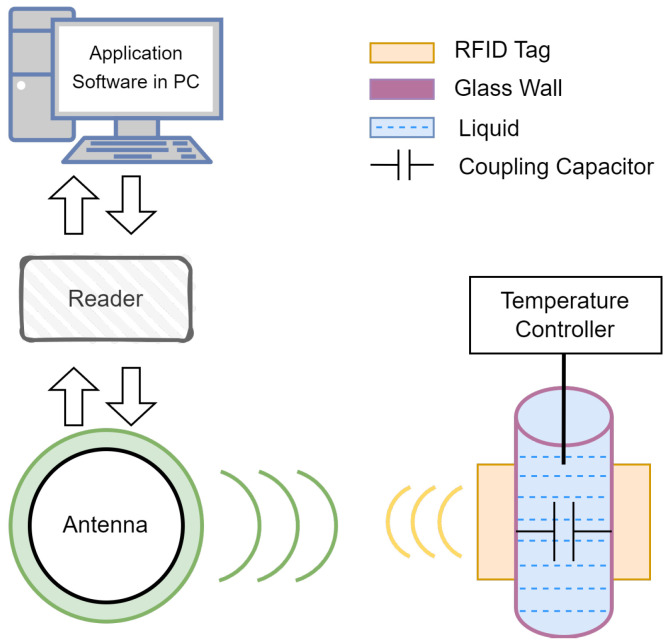
Block diagram of the wireless sensing system.

The proposed noninvasive sensing system consists of a working environment, a reader, and application software running on a personal computer (PC), as shown in Figure 1. The working environment includes dual-coupled tags, the test liquid, and the container. The reader comprises four parts: interface module, radio frequency (RF) module, control module, and power module. The interface module facilitates command interaction with the application software and data transmission. The control module serves as the core component, processing instructions and managing the working state of the entire reader. The RF module, connected to the transmission antenna, is responsible for awakening the passive tags and receiving data from them. The power module supplies energy to the entire system.

**Figure 2:**
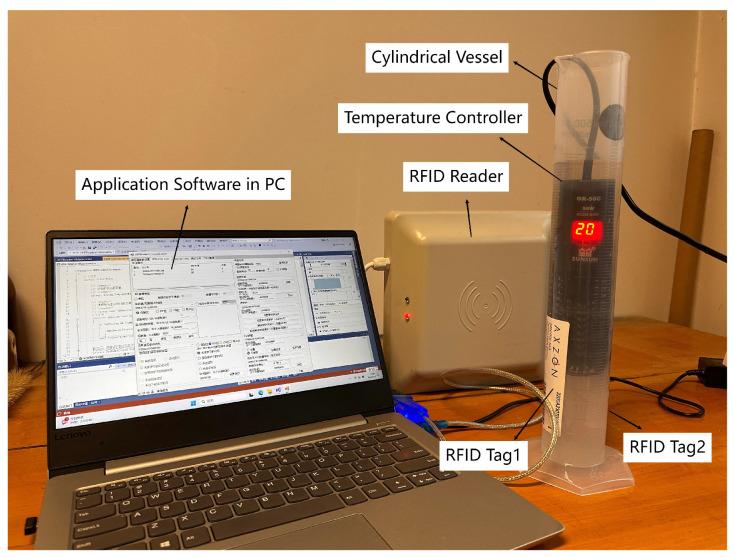
Actual experimental device.

The experimental device is shown in Figure 2. The RFID reader used in this work is the BY-RFID101 (Boyan Xintong Intelligent Technology Co., Ltd., Beijing, China). It is equipped with an 8 dBi circularly polarized antenna and provides a configurable read range of 0 to 6 m. The reader is connected to a PC via a USB-to-RS232 interface. The application software used is UHFReader18, which is specifically designed to work with the RFID101 reader. A cylindrical vessel serves as a container for the solution. Tags are attached to both sides of the container symmetrically. The size of the metal antenna part of the tag is 90 mm × 24.1 mm. The operating frequency range is 903–927.4 MHz. A temperature sensor maintains the solution at 20 °C during the experiment.

**Figure 3:**
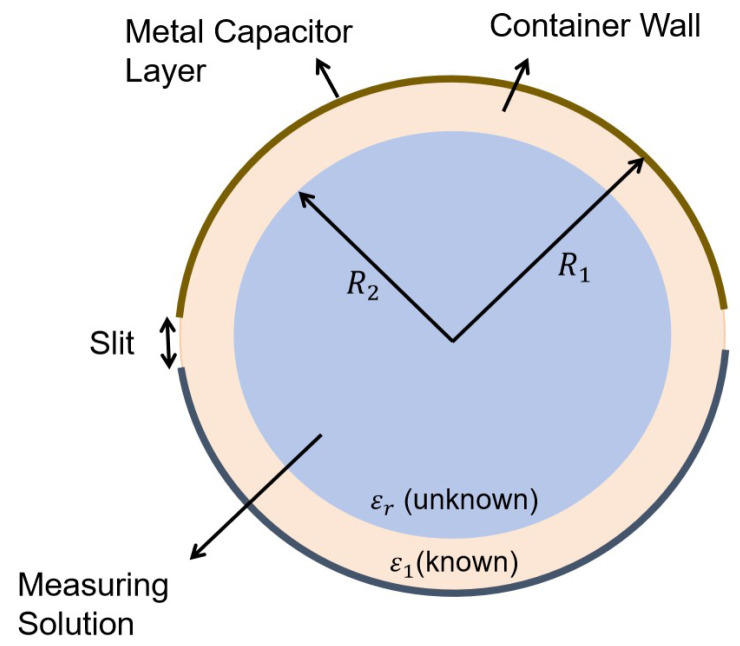
Section of the SCC model.

Changes in the concentration of the solution cause variations in the dielectric constant, typically exhibiting a nonlinear relationship, as the presence of the solute alters the polarization characteristics of the solution. According to capacitor theory, changes in the dielectric constant of the medium between the plates lead to changes in capacitance. The tags attached to the sides of the cylindrical container act as electrode plates, forming a coupling capacitance $C_{C}$. Therefore, $C_{C}$ can indirectly reflect changes in the concentration of the liquid. In pipeline environments, the slit cylindrical capacitance (SCC) model, as illustrated in Figure 3, was used in [16] to describe these scenarios. Figure 3 illustrates a top-down view of the container section from the experimental configuration depicted in Figure 2. The relationship between $C_{C}$ and permittivity can be expressed as

(1) $$C_{C}=\frac{2\varepsilon_{0}\varepsilon_{1}\varepsilon_{r}lR_{1}}{\sqrt{{\left(\varepsilon_{1}R_{2}\right)}^{2}-{\left(\varepsilon_{r}\left(R_{1}-R_{2}\right)\right)}^{2}}}\cdot \arctan\left(\sqrt{\frac{\varepsilon_{1}R_{2}-\varepsilon_{r}\left(R_{1}-R_{2}\right)}{\varepsilon_{1}R_{2}+\varepsilon_{r}\left(R_{1}-R_{2}\right)}}\tan\frac{\varphi }{2}\right)$$

where $\varepsilon_{0}$ is vacuum relative permittivity, $\varepsilon_{1}$ and $\varepsilon_{r}$ represent real-valued relative permittivity of the known and unknown dielectric layers, $R_{1}$ and $R_{2}$ are the radius of two dielectric layers, *l* is the length of the model cylinder, φ is central angle of arc, and $\varphi =\left(L_{l}/R_{1}\right)\in \left(0,\pi \right)$, where $L_{l}$ is tag arc length.

When $C_{C}$ changes, the impedance of the RFID tag antenna becomes mismatched with that of the chip. The UHF RFID tag with a built-in auto-tuning circuit can automatically adjust the matching capacitance $C_{M}$ to compensate for the impedance mismatch. As a result, the RFID reader can read $C_{M}$ as a direct measurement of the system. According to [17], the relationship between $C_{C}$ and $C_{M}$ can be expressed as (2)

(2) $$C_{M}=\frac{1}{Lw^{2}}-C_{C}+\frac{1}{rw}\sqrt{{\left(C_{C}rw\right)}^{2}-1}.$$

where *r* is built-in impedance, *L* is built-in inductance, ω is angular frequency, and ω=2πf, where *f* is frequency.

### 2.2. System Model

#### 2.2.1. State-Space Model Based on HMM

The Markov process is a fundamental stochastic model widely used for modeling dynamic systems with uncertainty. In this paper, a modified HMM is introduced to describe the concentration change in saline solution. Concentration and dielectric properties are considered as hidden states. $C_{M}$, obtained by the RFID sensing system, is treated as observation.

**Figure 4:**
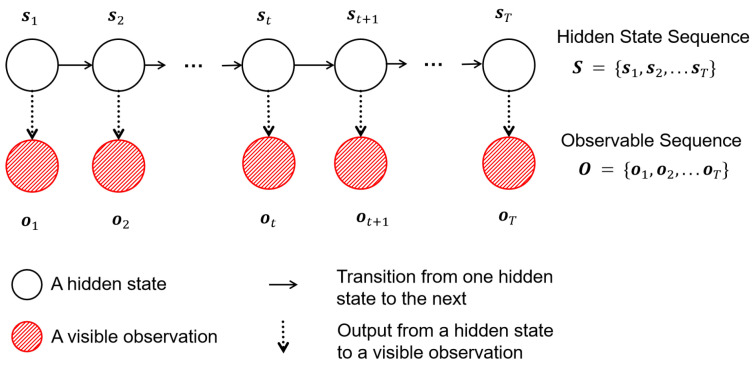
Relationship diagram of HMM.

Assuming that the hidden state of time *t* is only related to the hidden state of time t−1. From the hidden state at the previous time and the observation at the current time, the hidden state of the current time can be inferred. The relationship diagram is shown in Figure 4. As shown in Figure 4, the hidden state sequence is represented as $S=\{s_{1},s_{2},\ldots ,s_{T}\}$, where $s_{t}$ is the unobserved state at time *t*. The sequence follows the Markov property: $P\left(s_{t}|s_{t-1},s_{t-2},\ldots \right)=P\left(s_{t}|s_{t-1}\right)$. Observable Sequence is represented as $O=\{o_{1},o_{2},\ldots ,o_{T}\}$, where $o_{t}$ is the measured output at time *t*. Each hidden state $s_{t}$ emits an observation $o_{t}$ with probability $P(o_{t}|s_{t})$.

The state model can be expressed as

(3) $$s_{t}=f\left(s_{t-1},w_{t}\right)$$

where $s_{t}$ is current state, f(·) is state transition function, $w_{t}$ is process noise, and satisfies $w_{t}∼N\left(0,Q\right)$.

In order to obtain the concrete expression of the state transition function, we introduce the dielectric constant for theoretical analysis.

The Cole–Cole model is widely used to describe dielectric constant variation [18]. The generalized multirelaxation Cole–Cole model can be described as

(4) $$\varepsilon^{*}=\varepsilon^{\prime }-j\varepsilon^{\prime \prime }=\varepsilon_{\infty }\left(x\right)+\sum_{n=1}^{N}\frac{\Delta \varepsilon_{n}\left(x\right)}{1+{\left(jw\tau_{n}\left(x\right)\right)}^{1-\alpha }}+\frac{\delta_{0}}{jw\varepsilon_{0}}.$$

where $\varepsilon^{*}$ represents the complex permittivity to describe the dielectric properties of the medium. $\varepsilon^{\prime }$ and $\varepsilon^{\prime \prime }$ are the real and imaginary parts of $\varepsilon^{*}$, which represent the relative dielectric constant and the connection loss, respectively. *n* represents the order of the Cole–Cole model and *x* is concentration. $\varepsilon_{\infty }$ is the high-frequency limit permittivity, which represents the dielectric response of the material at high frequencies. ${\Delta \varepsilon }_{n}$ is the magnitude of dielectric relaxation. $\tau_{n}$ is the relaxation time constant, which describes the relaxation behavior of the material. α is the dielectric relaxation exponent, which adjusts the shape of the relaxation process. The value of α typically falls in the range of 0 to 1. Dielectric parameters ($\varepsilon_{\infty }$, ${\Delta \varepsilon }_{n}$ and $\tau_{n}$) are expressed as a function of concentration. In previous studies, researchers have found that dielectric properties are closely related to the concentration of electrolytes in the solution. Refs. [19,20] carried out dielectric spectrum measurement of chloride ion aqueous solution with different concentrations. These experimental data provide direct evidence for our proposed ionic dependence of dielectric parameters. In addition, from a theoretical perspective, the behavior of ions in solution affects the solvent molecules around them, thus changing the dielectric properties of the solution. This theoretical explanation also supports the idea of the ion dependence of the model parameters.

For analytical simplicity, the model can be reduced by neglecting the conductive loss term and assuming the presence of a single dominant relaxation process (N=1). Under these conditions, it simplifies to the standard Cole–Cole model:

(5) $$\begin{array}{c} \varepsilon^{*}=\varepsilon_{\infty }\left(x\right)+\frac{\Delta \varepsilon_{1}\left(x\right)}{1+{\left(j\omega \tau_{1}\left(x\right)\right)}^{1-\alpha }} \end{array}$$

The real part of $\varepsilon^{*}$, denoted as $\varepsilon^{\prime }$, can be calculated as

(6) $$\varepsilon^{\prime }=\varepsilon_{\infty }\left(x\right)+\frac{\Delta \varepsilon_{1}\left(x\right)\left[1+{\left(\omega \tau \left(x\right)\right)}^{1-\alpha }\sin\left(\frac{\pi \alpha }{2}\right)\right]}{1+2{\left(\omega \tau \left(x\right)\right)}^{1-\alpha }\sin\left(\frac{\pi \alpha }{2}\right)+{\left(\omega \tau \left(x\right)\right)}^{2(1-\alpha )}}$$

According to [21], the parameter α is set to 0.05 for salt solution. When α≈0, the term sin(πα/2) becomes negligibly small, and the real part of the Cole–Cole model can be approximated as

(7) $$\varepsilon^{\prime }\approx \varepsilon_{\infty }\left(x\right)+\frac{\Delta \varepsilon_{1}\left(x\right)}{1+{\left(\omega \tau \left(x\right)\right)}^{2(1-\alpha )}}$$

In the following analysis, only the real part $\varepsilon^{\prime }$ of the complex permittivity is considered. This simplification is justified because, in the frequency range of 903–927.4 MHz, the imaginary part $\varepsilon^{\prime \prime }$ is relatively small and exhibits limited variation with respect to solute concentration [19]. In contrast, $\varepsilon^{\prime }$ shows a more pronounced response to concentration changes and plays a dominant role in determining the capacitive behavior. Therefore, $\varepsilon^{\prime }$ provides sufficient information for accurate concentration estimation.

Assuming N=1. $\varepsilon_{\infty }\left(x\right)$, $\Delta \varepsilon_{1}\left(x\right)$ and $\tau_{1}\left(x\right)$ are dielectric parameters related to concentration. To establish the relationship between these parameters and concentration *x*, this article adopts a quadratic polynomial to fit $\varepsilon_{\infty }\left(x\right)$, $\Delta \varepsilon_{1}\left(x\right)$, and $\tau_{1}\left(x\right)$.

Assuming the estimated states to be $s={\left[\varepsilon^{\prime },x,\Delta \varepsilon_{1},\varepsilon_{\infty },\tau_{1}\right]}^{T}$. The state transition function can be expressed as

(8) $$\begin{array}{c} f\left(\cdot \right)=\left\{\begin{array}{c} {\left(\varepsilon^{\prime }\right)}_{t}={\left(\varepsilon_{\infty }\right)}_{t-1}+\frac{{\left(\Delta \varepsilon_{1}\right)}_{t-1}}{1+{\left(\omega {\left(\tau_{1}\right)}_{t-1}\right)}^{2(1-\alpha )}}\\ x_{t}=x_{t-1}\\ {\left(\Delta \varepsilon_{1}\right)}_{t}=a_{1}{\left(x_{t-1}\right)}^{2}+a_{2}x_{t-1}+a_{3}\\ {\left(\varepsilon_{\infty }\right)}_{t}=b_{1}{\left(x_{t-1}\right)}^{2}+b_{2}x_{t-1}+b_{3}\\ {\left(\tau_{1}\right)}_{t}=c_{1}{\left(x_{t-1}\right)}^{2}+c_{2}x_{t-1}+c_{3} \end{array}\right. \end{array}$$

where $a_{i},b_{i},c_{i}$ (i∈{1,2,3}) represent the coefficient to be fitted. Quadratic polynomials are introduced to fit the relationship between dielectric parameters and concentration due to their simplicity and effectiveness in capturing nonlinear trends. The dielectric properties of ionic solutions change nonlinearly with concentration. Quadratic polynomials provide a balance between model complexity and accuracy, enabling the description of these nonlinear dependencies with low computation complexity. Furthermore, previous studies [22] in related fields have demonstrated the feasibility of using quadratic polynomials to model similar relationships, achieving high fitting accuracy with minimal residual errors.

#### 2.2.2. RFID Observation Model

The observational model describes the relationship between the state of the system and the observed results, which can be expressed as

(9) $$z_{t}=h\left(s_{t-1}\right)+v_{t}$$

where h(·) is nonlinear observation function, and $v_{t}$ is RFID measurement noise and satisfies $v_{t}∼N\left(0,R\right)$.

In this study, the observed value of the RFID sensing system is $C_{M}$. Combined with (1), (2), and (6), the measurement model can be expressed as

(10) $$\begin{array}{cc} \; & h\left(s_{t}\right)=\frac{1}{L\omega^{2}}\\ \; & -\frac{2\varepsilon_{0}\varepsilon_{1}{\left(\varepsilon^{\prime }\right)}_{t}lR_{1}}{\sqrt{{\left(\varepsilon_{1}R_{2}\right)}^{2}-{\left({\left(\varepsilon^{\prime }\right)}_{t}\left(R_{1}-R_{2}\right)\right)}^{2}}}\cdot \arctan\left(\tan\frac{\varphi }{2}\sqrt{\frac{\varepsilon_{1}R_{2}-{\left(\varepsilon^{\prime }\right)}_{t}\left(R_{1}-R_{2}\right)}{\varepsilon_{1}R_{2}+{\left(\varepsilon^{\prime }\right)}_{t}\left(R_{1}-R_{2}\right)}}\right)\\ \; & +\frac{1}{r\omega }\sqrt{(\frac{2\varepsilon_{0}\varepsilon_{1}{\left(\varepsilon^{\prime }\right)}_{t}lR_{1}}{\sqrt{{\left(\varepsilon_{1}R_{2}\right)}^{2}-{\left({\left(\varepsilon^{\prime }\right)}_{t}\left(R_{1}-R_{2}\right)\right)}^{2}}}\cdot \arctan\left(\sqrt{\frac{\varepsilon_{1}R_{2}-{\left(\varepsilon^{\prime }\right)}_{t}\left(R_{1}-R_{2}\right)}{\varepsilon_{1}R_{2}+{\left(\varepsilon^{\prime }\right)}_{t}\left(R_{1}-R_{2}\right)}}\tan\frac{\varphi }{2}\right)r\omega {)}^{2}-1}. \end{array}$$

### 2.3. Algorithm Design for Concentration Tracking

Accurate tracking of salinity concentration in industrial wastewater using RFID measurements poses two key challenges: (1) the nonlinear relationship between RFID signal parameters and concentration, and (2) time-varying noise from turbulent flow and sensor fouling. To address these, we propose an improved EKF framework that linearizes the system dynamics locally while preserving the nonlinear observation model.

The EKF approximates nonlinearities by computing Jacobian matrices at each timestep. The state transition matrix at time *t* is approximated by the Jacobian matrix of (8) evaluated at time t−1, which can be expressed as

(11) $$\begin{array}{cc} F_{t}= & \left[\begin{array}{ccccc} 0 & 0 & \frac{1}{1+{\left(\omega {\left(\tau_{1}\right)}_{t-1}\right)}^{1-\alpha }} & 1 & \frac{2{\left(\Delta \varepsilon_{1}\right)}_{t-1}\left(1-\alpha \right){\left(\omega {\left(\tau_{1}\right)}_{t-1}\right)}^{1-2\alpha }}{{\left[1+{\left(\omega {\left(\tau_{1}\right)}_{t-1}\right)}^{2(1-\alpha )}\right]}^{2}}\\ 0 & 1 & 0 & 0 & 0\\ 0 & 2a_{1}x_{t-1}+a_{2} & 0 & 0 & 0\\ 0 & 2b_{1}x_{t-1}+b_{2} & 0 & 0 & 0\\ 0 & 2c_{1}x_{t-1}+c_{2} & 0 & 0 & 0 \end{array}\right] \end{array}$$

And the observational matrix is also approximate to a Jacobi matrix, which can be expressed as

(12) $$H_{t}=\left[\begin{array}{ccccc} \frac{\partial h(s_{t})}{\partial \epsilon^{\prime }} & 0 & 0 & 0 & 0 \end{array}\right]$$

The specific process of implementing hidden state estimation can be divided into two steps: prediction and updating. First, prediction step is realized according to Formula (3), which can obtain the prior estimation of the current state. Assuming that the distribution of hidden states satisfies: $s_{t-1}∼N\left(\mu_{t-1},P_{t-1}\right)$. After the prediction step, the prior distribution of hidden states at current time satisfies: $s_{t}∼N\left(\mu_{t|t-1},P_{t|t-1}\right)$. The prediction step can be expressed as

(13) $${\hat{s}}_{t|t-1}=f\left({\hat{s}}_{t-1|t-1},w_{t}\right)$$

(14) $$P_{t|t-1}=F_{t}P_{t-1|t-1}F_{t}^{T}+Q.$$

The update phase incorporates new measurements to refine the prediction. This Bayesian correction achieves minimum variance estimation through

(15) $$K_{t}=P_{t|t-1}H_{t}^{T}{\left(H_{t}P_{t|t-1}H_{t}^{T}+R\right)}^{-1}$$

(16) $${\hat{s}}_{t|t}={\hat{s}}_{t|t-1}+K_{t}\left(z_{t}-h\left({\hat{s}}_{t|t-1}\right)\right)$$

(17) $$P_{t|t}=\left(I-K_{t}H_{t}\right)P_{t|t-1}$$

In traditional EKF, R and Q are fixed, but in practice, variations in solution dynamics and RFID measurement conditions may cause time-varying noise statistics. Therefore, an adaptive adjustment strategy is introduced to improve the robustness of the system, which can be expressed as

(18) $$R_{t}=\alpha R_{t-1}+\left(1-\alpha \right)\left(\varepsilon_{t}\varepsilon_{t}^{T}+H_{k}P_{k}^{-}H_{k}^{T}\right)$$

(19) $$Q_{t}=\beta Q_{t-1}+\left(1-\beta \right)\left(K_{k}\varepsilon_{t}\varepsilon_{t}^{T}K_{k}^{T}\right)$$

where α and β are forgetting factor control the weight of historical information, and the innovation $\epsilon_{k}=z_{k}-h\left({\hat{s}}_{k}^{-}\right)$ reflects the discrepancy between measurements and predictions.

As shown in Algorithm 1, the modified AEKF is applied to the concentration tracking problem.
**Algorithm 1** Concentration estimation based on modified AEKF.**Initialization:**$P_{0},R_{0},Q_{0}$**Input:** measurement sequence $\left\{z_{t}\right\}$**Output:** real-time state estimate of the system $\left\{{\hat{s}}_{1},{\hat{s}}_{2},...,{\hat{s}}_{T}\right\}$, state estimation error covariance matrix $\left\{P_{1},P_{2},...,P_{T}\right\}$**for** k = 1 to T **do**   calculate ${\hat{s}}_{k|k-1}$ in (8)   calculate the Jacobian matrix $F_{k}$ in (11)   $P_{k|k-1}\leftarrow F_{k}P_{k-1}F_{k}^{T}+Q_{k}$   $K_{k}\leftarrow P_{k}^{-}H_{k}^{T}{\left(H_{k}P_{k}^{-}H_{k}^{T}+R_{k}\right)}^{-1}$   calculate $h({\hat{s}}_{k}^{-})$ in (10)   calculate observation matrix $H_{k}$ in (12)   ${\hat{s}}_{k}\leftarrow {\hat{s}}_{k}^{-}+K_{k}\left[z_{k}-h\left({\hat{s}}_{k}^{-}\right)\right]$   $P_{k}\leftarrow \left(I-K_{k}H_{k}\right)P_{k}^{-}$   update the covariance matrix $R_{k+1}$ and $Q_{k+1}$ using (18) and (19)**end for**

## 3. Experiments and Results

### 3.1. Sample Preparation

This article sets the concentration range of CaCl_2_ as 2–10 g/L. To ensure the accuracy of the concentration, the samples are prepared using CaCl_2_ (Fuchen (Tianjin) Chemical Reagent Co. Ltd., Tianjin, China) with a purity greater than 99.5%. Deionized water serves as the solvent. Different masses of CaCl_2_ solid are dissolved in deionized water to prepare samples of CaCl_2_ solutions with different concentrations.

### 3.2. Parameter Acquirement

Parameters $a_{i},b_{i},c_{i}$ in (8) are related to the solution type, reflecting the relationship between the dielectric constant and concentration. In order to establish the relationship in the modified HMM model, the following experiments are designed using an RFID sensing system.

Step 1: Prepare the samples and connect experimental equipment: Weigh the required mass of CaCl_2_ using an electronic scale (accuracy: 0.001 g). Prepare the CaCl_2_ solution in deionized water to the target concentration. Transfer the prepared solution into a cylindrical container, and insert a temperature controller into the solution and maintain the temperature at 20 °C. Connect the RFID reader to the PC and launch the UHFReader18 software. Operate the software to query the label and adjust the operating frequency. The operating frequency range is set to 903–927.4 MHz, with a step size of 0.4 MHz. Read and record the data at each frequency step using the software.

Step 2: Process data: In order to obtain the coefficient parameters, $\varepsilon_{\infty }\left(x\right)$, $\Delta \varepsilon_{1}\left(x\right)$, and $\tau_{1}\left(x\right)$ related to *x* are required. In order to fit (10), a new modified Levenberg–Marquardt method (NELM ) algorithm based on [23] is proposed. Specifically, the parameters to be fitted are expressed as $\psi =[\varepsilon_{\infty }\left(x\right),\Delta \varepsilon_{1}\left(x\right),\tau_{1}\left(x\right)]$. Then, the optimization problem is stated as follows:

(20) $$\psi^{*}=\arg\min_{\psi }\sum_{i}{\left(F\left(f_{i};\psi \right)-y_{i}\right)}^{2}=\arg\min r^{T}r$$

where *F* is model function, $f_{i}$ is sample frequency, and $y_{i}$ is the measured sample value of $C_{M}$ in different frequencies. $r={\left[r_{1},\ldots ,r_{s}\right]}^{T}$ is the residual vector and *s* is the data size. The iterative process is represented as follows:

(21) $$\begin{array}{cc} \; & \psi_{k+1}-\psi_{k}=J_{k}r\left(\psi_{k}\right)\\ & \cdot \left(J_{k}^{T}J_{k}+\left(\frac{\theta {∥r(\psi_{k})∥}^{\delta }}{1+{∥r(\psi_{k})∥}^{\delta }}+\frac{\left(1-\theta \right){∥J_{k}^{T}r\left(\psi_{k}\right)∥}^{\delta }}{1+{∥J_{k}^{T}r\left(\psi_{k}\right)∥}^{\delta }}\right)\mu_{k}I\right) \end{array}$$

where $J_{k}=\left[\left(\partial r/\partial \psi_{1}\right),\ldots ,\left(\partial r/\partial \psi_{t}\right)\right],\theta \in \left[0,1\right]$ and δ∈(0,1] are adjustable constants, $\mu_{k}$ is updated iteratively by the trust domain method, I is the identity matrix of order *s*, and *k* represents the *k*-th iteration. The direction and scope of each iteration is controlled by the trust region strategy:

(22) $$r_{k}^{*}=\frac{{\mathrm{ared}}_{k}}{{\mathrm{pred}}_{k}}=\frac{{∥r(\psi_{k})∥}^{2}-{∥r(\psi_{k+1})∥}^{2}}{{∥r(\psi_{k})∥}^{2}-{∥r\left(\psi_{k}\right)+J_{k}\left(\psi_{k+1}-\psi_{k}\right)∥}^{2}}$$

where, $r_{k}^{*}$ represents the approximation to objective. Set constant $p_{1},p_{2}\in \left(0,1\right)$, when $r_{k}^{*}<p_{1}$, $\mu_{k}$ should be raised; when $r_{k}^{*}>p_{2}$, $\mu_{k}$ should be reduced; and when $r_{k}^{*}\in \left[p_{1},p_{2}\right]$, no adjustment is required. Set the upper limit of the number of iterations $k_{max}$ and the upper limit of error *e*. The condition for ending iteration is that the number of iterations reaches $k_{max}$, or the mean relative error (MRE) is less than *e*.

According to the above method, the dielectric parameters are fitted using the $C_{M}$ measurement data of known concentration and frequency. Dielectric parameters and their corresponding concentrations are recorded.

Step 3: Determine parameters: Return to Step 1 and adjust the weight of CaCl_2_ to prepare solutions of different concentrations. Repeat Steps 2 and 3 to obtain multiple sets of dielectric parameters corresponding to these concentrations.

The data are then fitted using a quadratic polynomial to model the relationship between dielectric parameters and concentrations. The suitability of the fitting approach is based on previous experimental studies [22] and the physical nature of the system. Specifically, the dielectric parameters are influenced by ionic interactions and concentration-dependent effects, which exhibit nonlinear behavior under certain conditions. The quadratic polynomial provides a balance between model complexity and data accuracy for describing this relationship.

**Figure 5:**
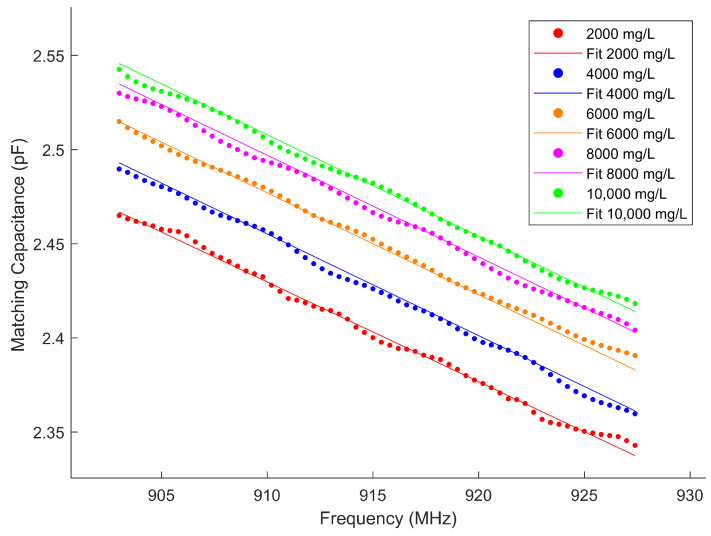
Experimental results and fitting results of different concentrations varying in frequency from 903 to 927.4 MHz.

The fitting parameters are summarized in Table 1, where $R^{2}$ represents the coefficient of determination, indicating the goodness of fit. The experimental and fitting results for different concentrations are illustrated in Figure 5, demonstrating the feasibility of this model. During the actual fitting process, we observe that the parameters $\Delta \varepsilon_{1}$ and $\tau_{1}$ exhibit minimal variations. Consequently, in the subsequent concentration tracking algorithm, these parameters are treated as constants.

### 3.3. Experimental Methods for Concentration Estimation

In order to verify the validity of the estimation model, CaCl_2_ solutions with known different concentrations are configured for the experiment.

Experiment 1: A set of CaCl_2_ solutions with increasing concentrations is prepared to simulate the rising pollutant concentration in wastewater. The concentration range is set from 2 g/L to 10 g/L, with a step of 1 g/L. For each concentration, the required mass of CaCl_2_ solid is accurately weighed using an electronic scale and dissolved in approximately 500 mL of deionized water. The solution is then transferred to a 1 L volumetric flask and diluted to the mark with deionized water to ensure consistent accuracy.

Capacitance measurements are carried out by the proposed wireless sensing system. At each concentration, the operating frequency of the system is swept from 903 MHz to 927.4 MHz, with a step size of 0.4 MHz. The measurement sequence consists of continuously updated capacitance values recorded across the specified frequency range.

Experiment 2: To demonstrate that the accuracy of the concentration estimation results is independent of time and that the algorithm can handle various concentration change trends, a new concentration sequence is designed. In this experiment, the concentrations do not follow a monotonic increase as in Experiment 1. Instead, the concentration sequence is set to alternate between increasing and decreasing values over time. This ensures that the algorithm is tested under dynamic conditions where the concentration changes unpredictably. The sequence includes a rearrangement of the same concentrations, and the corresponding capacitance values are recorded and analyzed using the same experimental setup as in Experiment 1. By comparing the estimated concentrations with the standard concentrations under this new sequence, we validate the algorithm’s ability to adapt to varying concentration trends and its independence from time-related changes.

In order to prove the applicability of this method for pipes of different materials and diameters, we designed Experiments 3 and 4. Since wastewater pipes in different companies vary, the diameter and material of the container pipe must be considered. The experiment involves four cylindrical containers of varying diameters, made of plastic (polypropylene) and glass. The diameters are summarized in Table 2.

Experiment 3: To demonstrate the influence of the container on measurement results, experiments are conducted using four cylindrical containers with varying diameters and materials (plastic and glass). The concentration of the CaCl_2_ solution is fixed, ensuring that any observed differences in measurement results can be attributed solely to the properties of the containers. By comparing the capacitance values obtained from each container, the experiment highlights the influence of container dimensions on the sensing performance and provides evidence for the need to recalibrate specific parameters when container properties change.

Experiment 4: To further test the robustness of the algorithm, a series of experiments with varying concentrations are conducted in Plastic Cylinder No. 3. The concentrations used in this experiment are 3000 mg/L, 5000 mg/L, 7000 mg/L, and 9000 mg/L. During this experiment, Algorithm 1 is applied to process the measurements in real time. This validates the algorithm’s ability to handle varying concentrations within a specific container while maintaining accurate estimation across a range of concentration values.

### 3.4. Experimental Results

To evaluate the performance of the model, MRE is applied to assess the model fitting and estimation performance, which can be calculated as

(23) $$MRE=\frac{100\%}{n}\sum_{i=1}^{n}\left|\frac{{\hat{y}}_{i}-y_{i}}{y_{i}}\right|$$

where ${\hat{y}}_{i}$ denotes the estimated output vector, $y_{i}$ denotes standard concentration, and *n* represents the number of samples. Relative error is calculated as

(24) $$e=\left|\frac{{\hat{y}}_{i}-y_{i}}{y_{i}}\right|$$

To evaluate fluctuations of the data at different concentrations, the standard deviation is introduced to describe the stability of the estimated data, as described in (25):

(25) $$u=\sqrt{\frac{\sum_{i=1}^{n}{\left({\hat{y}}_{i}-\bar{y}\right)}^{2}}{n-1}}$$

where $\bar{y}$ denotes the average of ${\hat{y}}_{i}$.

**Figure 6:**
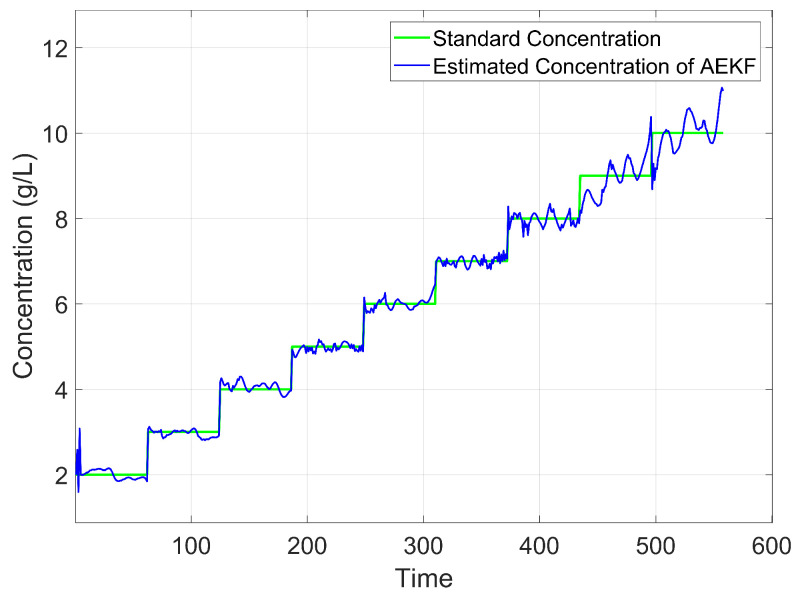
Comparison between estimated and standard concentration of CaCl_2_ solution.

**Figure 7:**
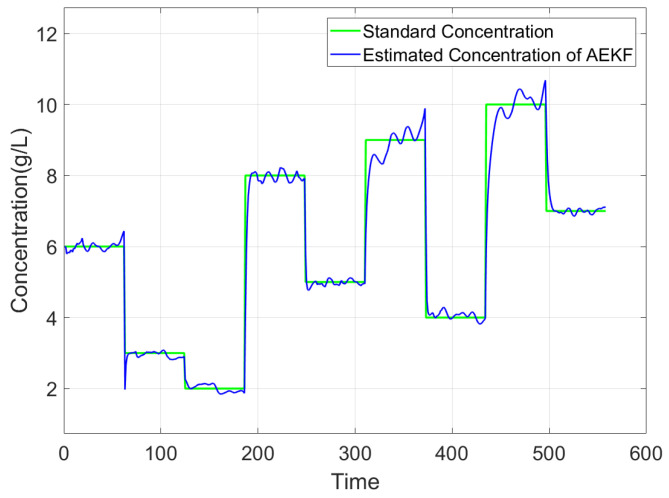
Comparison between estimated and standard concentration of CaCl_2_ solution in a new sequence.

The comparison between the estimated concentration and the standard concentration in Experiment 1 is illustrated in Figure 6. The standard concentration is obtained by weighing the solute using an electronic scale with a resolution of ±1 mg and diluting it to a specified total volume in a calibrated volumetric flask. Thus, the standard concentration in Figure 6 serves as a reliable reference for evaluating the estimated concentrations. It can be seen that the results demonstrate that the algorithm effectively tracks changes in concentration values. However, based on experimental findings, it is observed that after about 450 time units, the estimated concentration no longer exhibits significant step changes. To investigate whether this deviation is influenced by time or concentration variations, a comparison was made with Experiment 2. Figure 7 shows this set of concentration changes and the results of the algorithm monitoring. The results indicate that the sequence of concentration changes does not impact its monitoring outcomes and exclude the cumulative effect of time on the results. The method applies regardless of concentration changes. Similar to Experiment 1, instability in estimating concentrations occurs at levels around 9000–10,000 mg/L for Experiment 2.

To assess the concentration-dependent instability, Table 3 presents the statistical descriptors of the estimated concentrations in Experiment 1. The preparation error, originating from electronic scale measurements, is included in the analysis.

The results demonstrate that the standard deviation of the estimates is significantly higher at high concentrations. It can be seen that at 9000–10,000 mg/L concentration, the standard deviation exceeds 300 mg/L. At 2000–8000 mg/L, the standard deviation fluctuates around 100 mg/L. This significant increase in variability at elevated concentrations suggests that estimation instability becomes particularly pronounced in the high-concentration regime.

According to Experiment 1 and Experiment 2, we conclude that concentration is a contributing factor to these unstable estimates of the above experiments, suggesting the fitting parameters suit a certain range of applications. For CaCl_2_ solutions, the optimal application range is 2000–8000 mg/L. This result may be attributed to the inability of the current model to fully capture the dielectric properties at high concentrations. Furthermore, at elevated concentrations, the dielectric parameters exhibit saturation behavior, wherein changes in concentration result in diminishing variations in dielectric properties. This reduced sensitivity amplifies noise in the estimated concentrations, leading to the continuous noisy values observed in Figure 6 and Figure 7.

**Figure 8:**
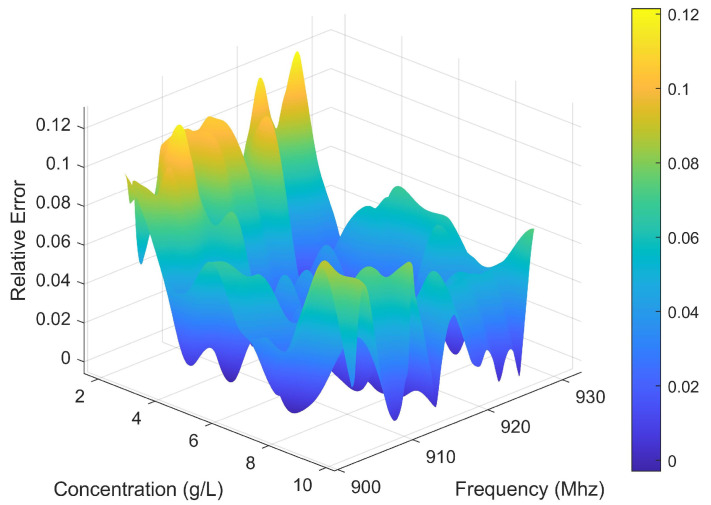
Relative error between the estimated concentration and the standard concentration.

In order to further determine the validity of the method, error analysis is performed. The MRE of Experiments 1 and 2 is 2.80% and 3.26%, respectively. Then, the rate of concentration change is varied to perform several experiments. The relative error is calculated according to the results of multiple experiments. Figure 8 shows the relative error between the estimated concentration and the standard concentration. The proposed framework effectively manages errors within a restricted range.

Experiments 1 and 2 are conducted in pipes of the same size, with results confirming the feasibility and accuracy of the proposed algorithm for estimating solution concentrations in wastewater pipelines. Building on these findings, Experiments 3 and 4 are designed to further evaluate the adaptability of the method across different pipeline sizes and materials, ensuring its applicability to diverse scenarios.

**Figure 9:**
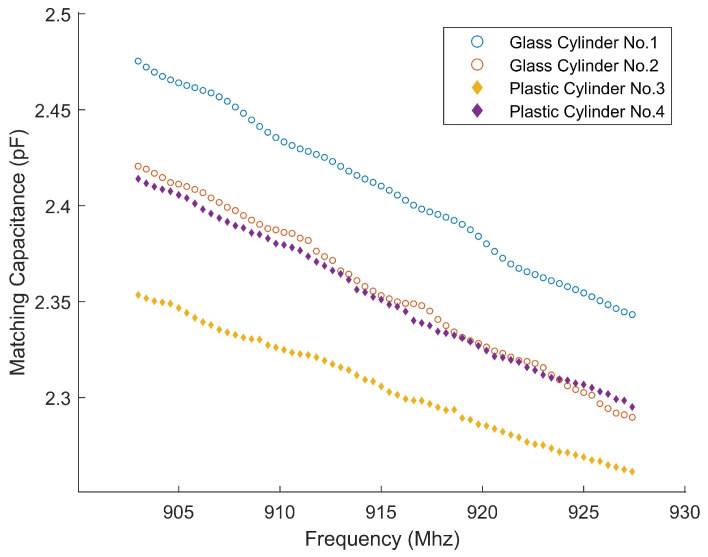
Comparison of experimental measurements of 3000 mg/L CaCl_2_ solution in different containers.

For Experiment 3, Figure 9 presents the comparison of experimental measurements of different containers at a fixed concentration of 3000 mg/L. Experimental results demonstrate that, for a fixed solution concentration, variations in container geometry and material lead to observable differences in the measured capacitance, indicating that the container properties exert a quantifiable influence on the sensing results. Moreover, despite these differences in measurement values, all container configurations exhibit a consistent frequency-dependent trend—a steady decrease in capacitance with increasing frequency. This consistent behavior suggests that the overall frequency response remains stable, confirming the feasibility and relative robustness of the proposed method when applied to containers of different types.

**Figure 10:**
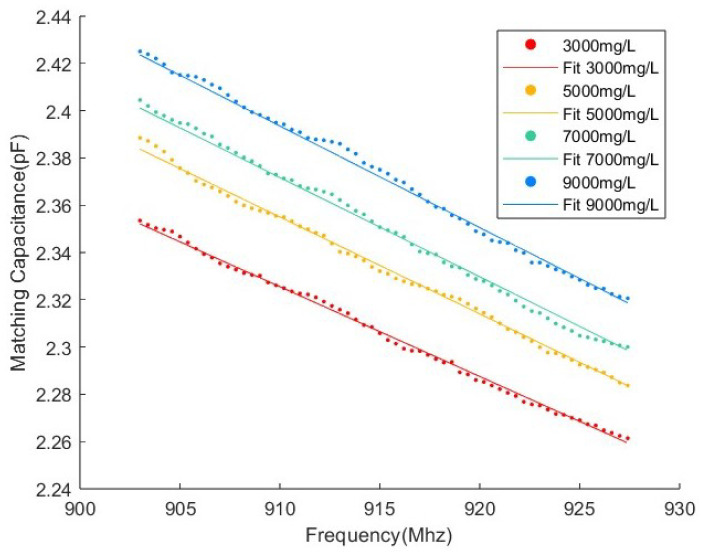
Experimental measurements and fitting measurements of different concentrations in Plastic Cylinder No. 3.

For Experiment 4, Figure 10 compares the experimental and fitted measurements at varying concentrations in Plastic Cylinder No. 3. Notably, these results demonstrate the same concentration-dependent trend as those obtained from Glass Cylinder No. 1 (Figure 5), confirming the model’s robustness across different container materials. This material independence establishes the feasibility of concentration estimation in plastic containers.

**Figure 11:**
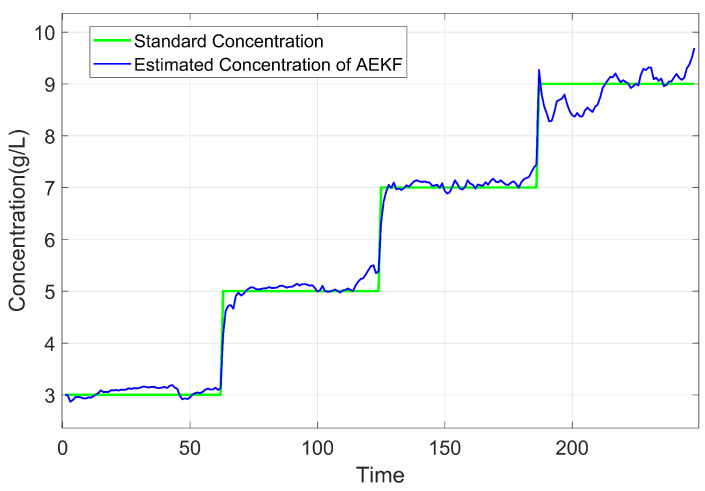
Comparison between estimated and standard concentration of CaCl_2_ solution in Plastic Cylinder No. 3.

**Figure 12:**
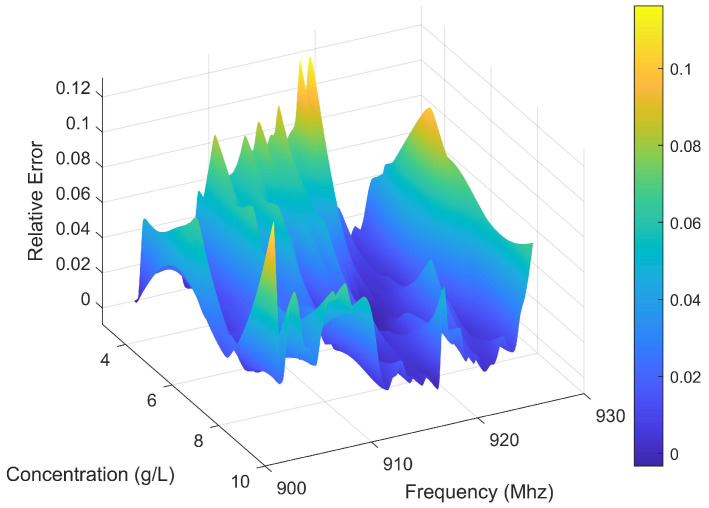
Relative error between the estimated concentration and the actual concentration in Plastic Cylinder No. 3.

Figure 11 presents the correlation between estimated and standard concentrations of CaCl_2_ solution in Plastic Cylinder No. 3. The data demonstrate that Algorithm 1 accurately tracks concentration variations in the plastic container, with container material showing negligible influence on estimation performance. Quantitative analysis reveals an MRE of 2.59%, with detailed error distribution shown in Figure 12.

Experiments 3 and 4 demonstrate the robustness and effectiveness of our proposed algorithm for various pipe sizes and materials. This adaptability highlights the versatility of the framework, ensuring its feasibility for diverse practical scenarios. These findings lay a strong foundation for the algorithm’s application within different wastewater companies, where pipe materials and sizes can vary significantly. Moreover, the consistent performance across different setups underscores the algorithm’s potential for large-scale implementation, providing a reliable solution for real-time monitoring and management of salt-containing wastewater.

### 3.5. Performance Evaluation

#### 3.5.1. Robustness Analysis Under Noise Conditions

To evaluate the algorithm’s robustness, we systematically design noise interference experiments by introducing different types of artificial noise into the measurements to analyze the algorithm’s concentration estimation performance under various noise conditions. Specifically, the experiments consider the following three typical noise models: (1) Gaussian Noise with σ = 0.15 pF, which can simulate random interference such as sensor thermal noise. (2) Impulse Noise composed of 95% Gaussian noise (σ = 0.15 pF) and 5% impulse (amplitude = 0.75 pF). This simulates occasional sensor faults or electromagnetic interference superimposed on baseline Gaussian noise. (3) Skewed Noise (Gamma distribution, shape parameter *k* = 2, σ = 0.15 pF). This represents asymmetric noise distributions common in electrochemical sensing systems.

**Figure 13:**
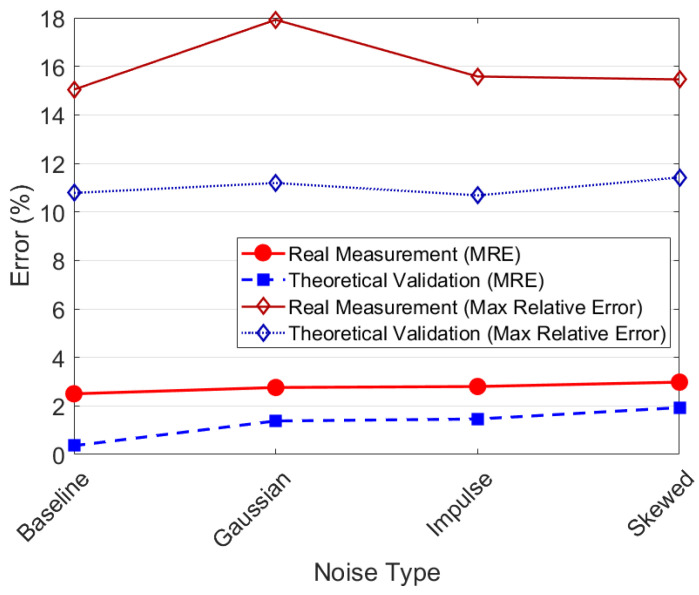
MRE and max relative error of real measurement experiments and theoretical verification experiments under different noise conditions.

**Figure 14:**
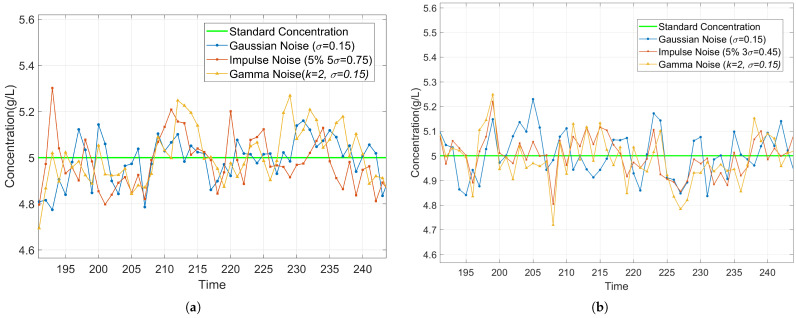
Comparison of Concentration Estimation under Artificial Noise. (**a**) Concentration Estimation with Added Noise Based on Real Sensor Data. (**b**) Concentration Estimation with Added Noise Based on Theoretical Data.

The experiments are divided into two types: real measurement experiments and theoretical validation experiments. In the real measurement experiments, noise is superimposed on the raw sensor data to evaluate the algorithm’s performance under practical noise conditions. On the other hand, in the theoretical validation experiments, measurement values are generated based on the ideal observation Equation (10), and noise is added, providing a theoretical benchmark for algorithm performance. According to the applicable range obtained from Experiment 1, the test subjects are CaCl_2_ solutions with concentrations ranging from 2 to 8 g/L. The MRE and max relative error of the two experiments are shown in Figure 13. Additionally, a subset of the concentration estimation results (5 g/L) is presented in Figure 14 to visually demonstrate the impact of different noise types.

The experimental results demonstrate that the system exhibits good robustness under various noise conditions. As shown in Figure 13, the MRE ranges from 2.50% to 2.98% for real measurements and from 0.11% to 1.82% for theoretical validation. The real measurement experiments exhibit higher baseline errors (MRE: 2.50%, Max: 15.04%) compared with theoretical validation (MRE: 0.11%, Max: 10.78%), reflecting inherent sensor noise and unmodeled environmental factors in practical implementations. When artificial noise is introduced, both experiments exhibit slight and stable increases in MRE. Figure 14 further presents the real-time estimation results at a concentration of 5 g/L. After adding noise to both the real measurements and theoretical values, the estimated concentrations stably fluctuate around the true values. In particular, impulse noise only causes transient spikes, and the system can quickly recover to a stable state. These results prove that the system has strong adaptability to Gaussian noise, impulse noise, and skewed noise, maintaining stable detection performance in both theoretical simulations and practical applications.

#### 3.5.2. Computational Efficiency Comparison

To systematically evaluate the comprehensive performance of AEKF in concentration tracking tasks, comparative experiments are conducted. The Unscented Kalman filter (UKF) and Particle filter (PF) are selected as baseline methods, where the UKF represents nonlinear filtering approaches based on deterministic sampling, while the PF serves as the benchmark algorithm employing Monte Carlo simulation. The number of particles in PF is set to $n_{p}=2000$. Experiments are conducted using CaCl_2_ solutions with concentrations ranging from 2 to 8 g/L, comprising a total of 434 data samples. The algorithms are evaluated in terms of estimation accuracy and computational efficiency. Estimation accuracy is assessed using the MRE as the performance metric. Computational efficiency is measured by execution time under identical hardware conditions. The evaluation is performed using the tic-toc function in MATLAB R2019b (The MathWorks, Inc., Natick, MA, USA), averaged over 100 iterative runs.

**Figure 15:**
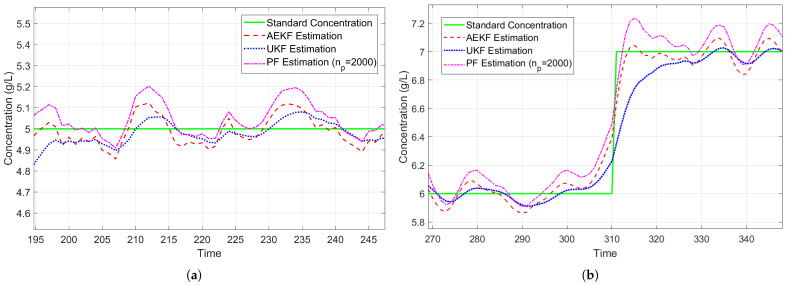
Comparison of concentration estimation results of AEKF, UKF, and PF. (**a**) The estimation result of CaCl_2_ concentration at a concentration of 5 g/L. (**b**) The estimation result of CaCl_2_ concentration when the concentration changes from 6 g/L to 7 g/L.

Figure 15 presents the estimation results of AEKF and baseline algorithms in the CaCl_2_ solution concentration estimation experiment. As shown in Figure 15, all algorithms are capable of tracking the dynamic variation trends of the standard concentration, while exhibiting distinct performance characteristics. The UKF produces smoother concentration curves but exhibits noticeable lag during concentration changes. The PF responds more quickly to concentration variations but demonstrates higher fluctuations. Notably, the proposed AEKF maintains high estimation accuracy while achieving faster response times, making it beneficial for dynamic concentration tracking applications.

Table 4 summarizes the MRE and average execution time per iteration. As shown in Table 4, the AEKF outperforms other algorithms in terms of both estimation accuracy and computational efficiency. These results confirm the effectiveness of AEKF in moderately nonlinear systems, where its first-order approximation framework maintains accuracy while achieving computational efficiency. Although UKF is theoretically more suitable for strongly nonlinear systems, it did not demonstrate significant advantages in this experiment. Due to its high computational complexity, the PF algorithm requires substantially longer execution time, making it unsuitable for applications with stringent real-time requirements.

## 4. Discussion

### 4.1. Discussion of Experimental Findings

Experimental results demonstrate an optimal detection range of 2000–8000 mg/L for CaCl_2_ solutions using our proposed method. This phenomenon may be attributed to the model’s inability to fully capture dielectric properties at high concentrations. In practical applications, this effective concentration range sufficiently meets research needs. Our optimal range (2000–8000 mg/L) is compatible with wastewater treatment targets. In the real wastewater treatment process, salt concentrations exceeding 8500 mg/L significantly disrupt wastewater treatment processes, including microbial activity and phosphorus removal efficiency, while lower concentrations (≤8000 mg/L) have minimal impact on system performance [24,25]. These insights reinforce the practical significance of monitoring salt concentrations within the 2000–8000 mg/L range.

The experimental setup includes both plastic and glass cylindrical containers of varying sizes, demonstrating the system’s adaptability to different container specifications. Notably, the proposed method achieves an MRE as low as 2.59% under the conditions of Experiment 4 with plastic cylinders, further validating its robustness and practical applicability across diverse real-world scenarios.

While this study demonstrates promising results, certain limitations should be considered for practical applications. The experimental validation focused on controlled CaCl_2_ solutions, whereas real wastewater may contain additional salts (e.g., NaCl and MgSO_4_) and organic compounds whose potential interference requires further investigation. In future studies, we will validate the system using synthetic wastewater mixtures to evaluate its performance under more realistic conditions.

A comparison with several representative noninvasive concentration sensing techniques is provided in Table 5. The Ultraviolet–visible spectroscopy (UV-Vis) approach offers high accuracy (MRE = 0.63%), but it typically requires optical alignment and transparent pipelines, and is sensitive to interference from turbidity and color variations. Due to these requirements, it is generally unsuitable for real-time applications. Ultrasound sensing can achieve through-wall measurements, yet tends to exhibit relatively large errors and demands precise transducer alignment. In enclosed containers, multiple reflections can cause echo overlap, further affecting the measurement accuracy. Microwave microfluidic sensors demonstrate excellent resolution across a wide concentration range (0.4 g/L), but their implementation relies on microfabrication and high-frequency electronics, resulting in increased system complexity and cost.

In contrast, the RFID-based system proposed in this work provides a low-cost, low-complexity solution for measuring CaCl_2_ concentrations in the 2–10 g/L range. Although its MRE is slightly higher than that of optical methods, the simplicity and robustness of the RFID system make it particularly suitable for on-site deployment and long-term monitoring in corrosive environments.

### 4.2. Practical Considerations and Future Improvements

The proposed RFID-based sensing system performs reliably in controlled conditions and lays a solid foundation for noninvasive concentration monitoring. To extend its application in real-world pipeline environments, several factors warrant consideration.

Variations in temperature may introduce dielectric drift; however, in our current experiments, this influence was minimized through strict temperature control. To further investigate the impact of temperature fluctuations, additional experiments are planned under varied temperature conditions. For future field deployment, such effects could be mitigated through compensation algorithms or real-time temperature monitoring.

Long-term usage may also encounter salt accumulation near the antenna region, potentially altering the local dielectric properties. Although this was not observed within the duration of our experiments, industrial applications may require periodic cleaning or the use of hydrophobic coatings to reduce inner wall contamination.

Additionally, fluid motion may cause measurement variability, serving as a source of dynamic noise. The influence of flow rate on measurement performance will be systematically explored in future studies.

## 5. Conclusions

This study develops a novel RFID sensing system integrated with an AEKF algorithm for noninvasive, real-time monitoring of CaCl_2_ solution concentrations. By combining dual-tag RFID architecture with physics-based Cole–Cole and SCC modeling, we establish the state model and observation model for the proposed RFID system. Experimental results indicate that the system achieves an MRE of 2.80% across CaCl_2_ solutions spanning 2000–10,000 mg/L, with an optimal application range of 2000–8000 mg/L. AEKF algorithm successfully addresses the challenges of nonlinear observation models and time-varying noise through its recursive estimation framework and adaptive covariance tuning. Experiment results demonstrate that AEKF is robust to noise and has significant improvements over baseline methods in both accuracy and computational efficiency.

Furthermore, the system maintains reliable performance across different container materials and sizes, with MRE as low as 2.59% in plastic cylindrical containers, confirming its adaptability for practical deployment. Future work will expand validation to synthetic and actual wastewater. These advancements will further bridge the gap between laboratory prototypes and industrial deployment, particularly in wastewater treatment and process control scenarios.

## Figures and Tables

**Table 1:** Quadratic fitting coefficient of CaCl_2_.

Parameter	$k_{1}$	$k_{2}$	$k_{3}$	$R^{2}$
$\Delta \varepsilon_{1}$	$a_{1}=-9.816\times 10^{-4}$	$a_{2}=0.0208$	$a_{3}=0.987$	0.9980
$\varepsilon_{\infty }$	$b_{1}=0.0177$	$b_{2}=3.1583$	$b_{3}=8.4875$	0.9974
$\tau_{1}$ (ns)	$c_{1}=2.892\times 10^{-5}$	$c_{2}=-6.24\times 10^{-4}$	$c_{3}=35$	0.9982

**Table 2:** Changed Parameters for Different Containers in Experiment 3 and Experiment 4.

Container	$R_{1}$ (mm)	$R_{2}$ (mm)
Glass Cylinder No. 1	12.45	10.9
Glass Cylinder No. 2	21	19
Plastic Cylinder No. 3	28	25
Plastic Cylinder No. 4	36	34

**Table 3:** Error analysis for each concentration in Experiment 1.

Standard Concentration (mg/L)	Preparation Error (%)	Average Estimated Concentration (mg/L)	Standard Deviation of Estimated Concentration (mg/L)	MRE (%)
2000	0.50	2015.7	193.5	6.13
3000	0.33	2958.3	83.9	2.44
4000	0.25	4061.7	115.3	2.71
5000	0.14	4966.1	94.0	1.65
6000	0.12	6006.7	136.8	1.71
7000	0.14	6997.6	102.4	1.19
8000	0.12	7968.4	161.0	1.67
9000	0.11	8953.6	467.5	4.16
10,000	0.10	9996.9	467.4	3.51

**Table 4:** Performance comparison of the proposed algorithm and the baseline algorithm.

Algorithm	MRE (%)	Execution Time (ms)
AEKF	2.50	151.35
UKF	2.70	293.36
PF	2.58	2018.37

**Table 5:** Comparison of noninvasive concentration sensing methods.

Method	Solutions	Concentration	Performance	Real-Time
[26] (UV-Vis)	Water/Cu^2+^	0.1–7.7 g/L	MRE = 0.63%	No
[27] (Ultrasound)	Water/Solid	0.21–1.24% (Volume)	MAE = 2.72–6.85%	Yes
[28] (Microwave)	Water/Glucose	0.3–80 g/L	Resolution = 0.4 g/L	Yes
This work (RFID)	Water/CaCl_2_	2–10 g/L	MRE = 2.80%	Yes

## Data Availability

The original contributions presented in this study are included in the article. Further inquiries can be directed to the corresponding author.
